# Sonoporation-mediated gene transfer into adult rat dorsal root ganglion cells

**DOI:** 10.1186/1423-0127-17-44

**Published:** 2010-06-03

**Authors:** Chung-Ren Lin, Kuan-Hung Chen, Chien-Hui Yang, Jiin-Tsuey Cheng, Shyr-Ming Sheen-Chen, Chih-Hsien Wu, Wei-Dih Sy, Yi-Shen Chen

**Affiliations:** 1 Department of Anesthesiology, Chang Gung Memorial Hospital-Kaohsiung Medical Center, Chang Gung University College of Medicine, Kaohsiung, Taiwan; 2 Department of Surgery, Chang Gung Memorial Hospital-Kaohsiung Medical Center, Chang Gung University College of Medicine, Kaohsiung, Taiwan; 3 Department of Anesthesiology, National Taiwan University College of Medicine, Taipei, Taiwan; 4 Department of Biological Sciences, National Sun Yat-Sen University, Kaohsiung, Taiwan

## Abstract

**Background:**

Gene transfer into many cell types has been successfully used to develop alternative and adjunct approaches to conventional medical treatment. However, effective transfection of postmitotic neurons remains a challenge. The aim of this study was to develop a method for gene transfer into rat primary dorsal root ganglion neurons using sonoporation.

**Methods:**

Dissociated cells from adult rat dorsal root ganglion (DRG) cells were sonicated for 1-8 s at 2.5-10 W to determine the optimal ultrasound duration and power for gene transfection and cell survival. Transfection efficiency was compared between sonoporation, liposome and lentiviral vector gene transfer techniques.

**Results:**

The optimum ultrasound intensity was 5 W for 2 s and yielded an efficiency of gene transfection of 31% and a survival rate of 35%.

**Conclusions:**

Sonoporation can be optimized to minimize cell death and yield a high percentage of transfected neurons and that this technique can be easily applied to primary cultures of rat dorsal root ganglion neurons.

## Background

Methods for altering gene expression are widely used to elucidate molecular mechanisms involved in cellular physiopathology. Gene modification also has potential as a therapeutic modality for treating many diseases. Many gene transfer methods have been developed in the past two decades, including calcium phosphate coprecipitation, microinjection, recombinant viruses, liposome-mediated gene transfer, lipids that do not form liposomes, high molecular weight cationic polymers, particle bombardment (biollistics) and electroporation.

Ultrasound-mediated gene transfer has recently emerged as a promising technique with a broad range of potential applications. Ultrasound can be used to modify the permeability of the cell membrane to facilitate the uptake of RNA [1-5] and DNA into the cell [6-11]. Low-frequency ultrasound increases membrane permeability to many drugs, including high molecular weight proteins [12]. The degree of macromolecule uptake is correlated with the acoustic energy and frequency of the stimulus [13]. Miller et al. [14] demonstrated that the uptake of fluorescent dextran by Chinese hamster ovary cells is similar at ultrasound frequencies of 1.0 MHz and 3.3 MHz, but is greatly reduced at 5.3 MHz and 7.15 MHz. Huber and Pfisterer reported that focused ultrasound enhanced the transfer of DNA plasmids into several cell lines *in vitro *and into a Dunning prostate tumor after direct DNA injection *in vivo*. They showed that the pressure amplitude and duration of sonication affect transfection ratio and cell survival [15].

Transfection of postmitotic neurons has been a major challenge in the past. With few exceptions, neuronal transfections have been unreliable, cytotoxic, labor-intensive and inefficient [16]. Although sonoporation-mediated gene transfer into cell lines [8,17,18], silkworm larvae [19], the ovary and uterus [20], muscle cells [21], the salivary gland [22], the joint synovium [4] and the chicken embryo [23] has been described, there are few reports on sonoporation-mediated gene transfer into neuronal cells. Shimamura et al. used ultrasound and microbubble-mediated cavitation to transfect rat brain cells [24]. They successfully transfected meningeal and glial cells, but failed to transfect neurons. Manome et al. reported that they transfected neuronal cells in brain slices, but in very small amounts [7]. Fischer et al. used sonoporation to transfect plasmids into the retinal neurons, dorsal forebrain and optic tectum of the chicken, into the cerebellar neurons of the rat and into the hippocampal neurons of the mouse. Although the ability of ultrasound to enhance gene transfer has been demonstrated in numerous studies, its efficacy for gene transfer into the dorsal root ganglion (DRG) has not been determined.

This study was designed to investigate the feasibility of gene transfer into the DRG using a standard laboratory sonicator and to determine the optimal ultrasound duration and power for gene transfection and DRG survival. We also compared transfection (transduction) efficiency between the sonoporation, liposome and lentiviral vector gene transfer techniques.

## Materials and methods

### Animals

The animals were used in accordance with the guidelines of the Chang Gung University.

### Tissue culture

DRG cells were prepared as previously described [25]. DRGs were dissected in sterile Hanks' buffered saline solution (HBSS) containing 3% D-glucose and 0.01 M HEPES buffer (HBSS+). DRG cells were dissociated by mild trituration using a Pasteur pipette after incubation for 10 min at 37°C in Ca^2+^/Mg^2+^-free HBSS containing 0.05% trypsin. The cell suspensions were initially plated into noncoated plastic tissue culture flasks and left for 3 h at room temperature to minimize the number of fibroblasts in the cultures. The supernatant, which contained DRG neurons and satellite glial cells, was then aspirated. Cell density was determined using a hemacytometer. Between 100,000 and 200,000 cells were plated onto 12 mm glass cover slips that were coated sequentially with poly-D-lysine and laminin (Sigma) diluted 1:100 in HBSS. Cell cultures were maintained at 37°C under a 5% CO_2 _atmosphere in culture medium (Neurobasal Media, Gibco) containing N-2 supplement (Gibco), 100 U/ml penicillin, 100 mg/ml streptomycin and 2% fetal bovine serum (Gibco BRL).

### Virus preparation

Enhanced green fluorescence protein (E-GFP)-containing lentiviral vectors were obtained from the National RNAi Core Facility, Academia Sinica, Taiwan. Lentiviruses were prepared according to a standard protocol. Titers were assayed using 3T3 cells and serial dilutions of the vector preparations. The titers of these vector stocks were also estimated by measuring the level of viral p24gag antigen using a human immunodeficiency virus-1 p24 antigen assay kit (Beckman Coulter, Fullerton, CA).

### Liposome-mediated gene transfer

Lipofectamine 2000 (Invitrogen) was mixed with plasmid E-GFP DNA (pE-GFP C1) in the ratio of 3 μl per 2 μg, respectively, in 50 μl of HBSS+. The vector is described elsewhere [26]. The optimum ratio of lipofectamine to pE-GFP C1 was determined according to the manufacturer's instructions. These reagents were incubated at room temperature for 15 min and were then added to a solution containing freshly dissected DRG cells. The medium was replaced with Lipofectamine 2000 after 12 h.

### Sonoporation

We used a Sonics and Materials VC130 sonicator (Newtown, CT), which produces continuous wave ultrasound at 20 kHz and has an adjustable output range of 2.5-130 W and a 12 mm diameter probe tip. pE-GFP C1 was diluted in HBSS+, added to the wells at concentrations of 0.5-20 μg/ml and incubated for 5-10 min at 37°C. The sonicator probe was sterilized by sonication in 70% ethanol followed by sonication in sterile water. It was then placed into the wells, each of which contained 1000 μl of medium, and activated for 1-8 s, which delivered 2.5-10 W of energy. Cells were cultured for 1-14 d after sonoporation and were then fixed and processed for immunocytochemical labeling using standard methods [27].

### Cell survival assay

Cell survival after sonoporation was assessed using the Live/Dead assay (Molecular Probes) according to the manufacturer's specifications. DRG cells were maintained *in vitro *for 48 h after sonication. The medium was then aspirated and cells were rinsed twice with HBSS+. After adding 200 μl of HBSS+ containing 1 μM ethidium homodimer and 1 μM calcein AM, the cells were incubated for 30 min at 37°C, rinsed twice in HBSS+ and counted using a fluorescence microscope.

### Fixation and immunocytochemistry

Cells were fixed and immunolabeled as described previously [27]. The primary antibodies were mouse anti-β-tubulin isotype III (1:1000; Sigma) and rabbit anti-E-GFP (1:1000; Sigma). The secondary antibodies were goat anti-rabbit Alexa568, goat anti-mouse Alexa568 and goat anti-rat Alexa488 (Molecular Probes Inc., Eugene, OR) and were diluted 1:1000 in PBS containing 0.2% Triton X-100. Cells were stained using 1 μg/ml of DAPI (Sigma) in PBS and then mounted on cover slips using Fluoromount-G.

### Statistical analysis

Means for experimental variables were compared using ANOVA and Student's *t*-test post hoc. Means and standard errors for cell numbers were calculated by counting cells in at least five view fields from at least three cover slips per treatment.

## Results

### The optimal power for sonoporation of DRG cells is 5 W

**Figure 1:**
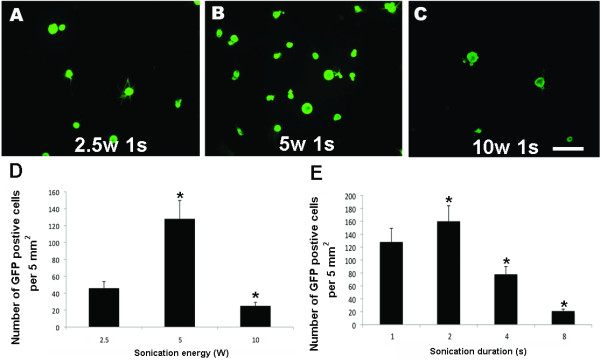
**Optimal conditions for DRG transfection via sonoporation**. Dissociated cells from adult rat DRGs were suspended at a density of 10,000 cells per cm^3 ^in a solution containing 10 μg/ml of pE-GFP C1 and were sonicated for 1 s at 2.5-10 W using a 12 mm diameter probe tip. The cells were maintained *in vitro *for 48 h after sonoporation. The calibration bar in Panel c represents 100 μm and applies to Panels a-c. Panel d shows the number of GFP-expressing cells per 5 mm^2 ^after sonication at various energy levels. Panel e shows the number of GFP-expressing cells per 5 mm^2 ^after sonication for various sonication durations at 5 W.

**Figure 2:**
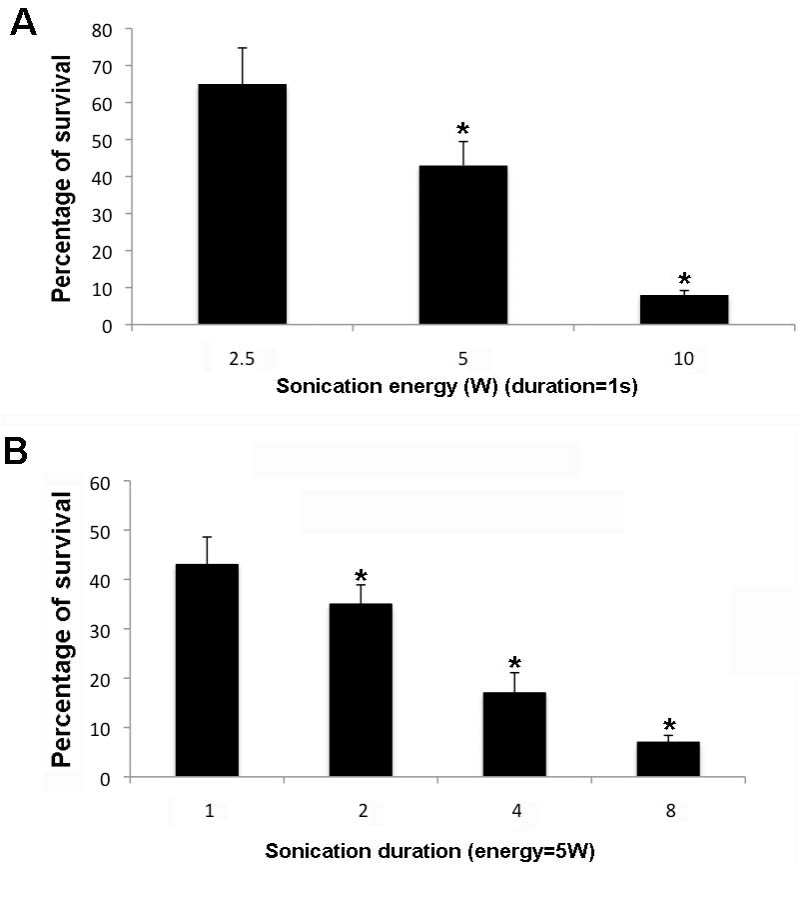
**Cell survival for various sonoporation durations and energy levels**. Survival was defined as the number of calcein-positive cells 2 d after sonoporation and expressed as a percentage of the number of calcein-positive cells in the unsonoporated control. Results are expressed as the mean ± SD. *p < 0.05.

We conducted a series of experiments using cultures of DRG cells to determine the optimal energy output for sonoporation. Dissociated cells from adult rat DRGs were suspended at a density of 10,000 cells per cm^3^. After addition of 10 μg/ml of pE-GFP C1, the suspensions were sonicated for 1 s at 2.5-10 W. The cells were maintained *in vitro *for 48 h after sonoporation. GFP expression showed that a few cells were transfected by treatment for 1 s at 2.5 W (Figure 1A). A twofold increase in output energy from 2.5 W to 5 W increased the number of GFP-positive cells by threefold (Figure 1B). However, the number of GFP-positive cells decreased when the output energy was increased further to 10 W (Figure 1C), probably because of decreased cell survival (Figure 2). The optimal output energy for sonoporation for 2 s was 5 W according to the number of GFP-positive cells (Figure 1D).

### The optimal duration of sonoporation of DRG cells is 2 s

To determine the optimal duration of sonoporation, we conducted another series of experiments using DRG cells. Dissociated cells from adult rat DRGs were suspended at a concentration of 10,000 cells per cm^3 ^and 10 μg/ml of pE-GFP C1 was added to the medium. The suspensions were then sonicated at 5 W for 1-8 s. The cells were maintained *in vitro *for 48 h after sonoporation. The number of GFP-positive cells increased up to a sonoporation duration of 2 s (Figure 1E).

### The survival rate of DRG cells after sonoporation for 2 s at 5 W is 35%

The number of dead cells after sonoporation was assessed according to the accumulation of ethidium homodimer and the number of live cells was assessed according to the accumulation of calcein AM. The number of dead cells after sonication for 1 s at 2.5 W was 35% of that of the unsonicated control. Sonication for 1 s at 5 W or 10 W increased the number of dead cells to 43% of that of the control. At 5 W, sonication for 2 s, 4 s and 8 s increased the number of dead cells to 65%, 87% and 93%, respectively, of that of the control (Figure 2).

### Comparison of transfection (transduction) efficiency between the sonoporation, liposome and lentiviral vector methods

**Figure 3:**
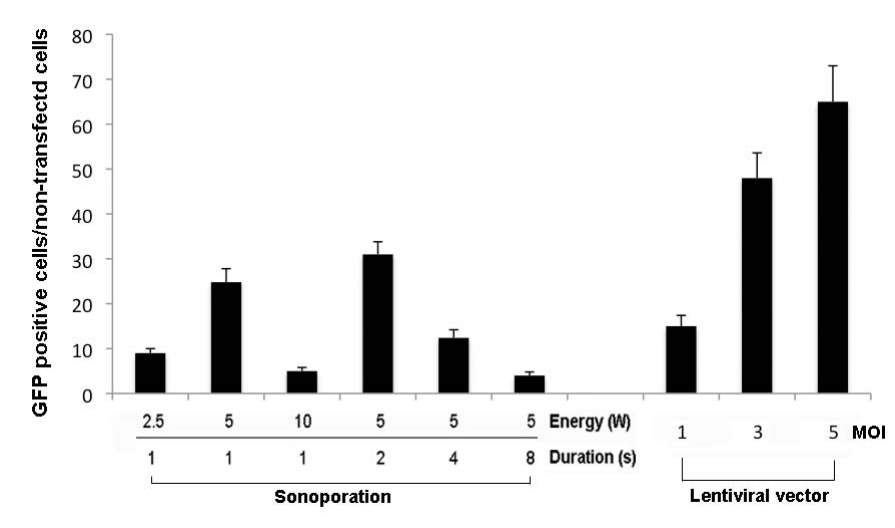
**Comparison of gene transfection efficiency between the sonoporation and lentiviral vector methods**. The histogram shows the relative number of transfected GFP-positive cells per 5 mm^2 ^after sonoporation (left side of the panel) and gene transfer using the lentiviral vector (right side of the panel). All DRG cells were derived from the same pool of cells.

Cells used for these experiments were obtained from the pooled dissociated adult rat preparations before they were allocated to the free-floating and adherent-cell treatments. Less than 1% of cells transfected with liposomes were GFP-positive (data not shown). Lentiviral vectors added at MOIs of 1, 3 and 5 transduced 10%, 48% and 65%, respectively, of the DRG cells (Figure 3). Although the efficiency of the lentivirus method was much higher than that of the sonoporation method, preparation of the viral vector was much more labor-intensive than the sonoporation method.

### Percentage of neuronal cells transfected using sonoporation

**Figure 4:**
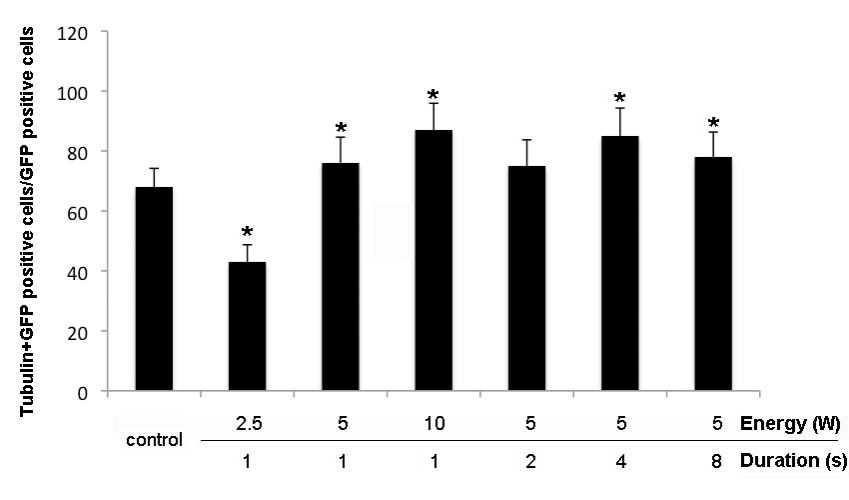
**β-tubulin III- and GFP-immunoreactive DRG cells**. After addition of 10 μg/ml of pE-GFP C1, the cells were sonicated for 2 s at 5 W using a 12 mm probe. The cells were processed for immunocytochemistry 48 h after sonoporation and labeled with antibodies against GFP and β-tubulin III (a marker of neuronal cells).

We used immunocytochemistry against β-III tubulin to identify neuronal cells in primary DRG cultures incubated with 10 ng/ml of NGF for 48 h. The percentage of β-III tubulin-positive cells in the control culture was 68.0 ± 6.2%. After sonoporation for 1 s at energy outputs of 2.5 W, 5 W and 10 W, cells immunoreactive for β-III tubulin constituted 43%, 76% and 87%, respectively, of GFP-transfected cells. At an energy output of 5 W and sonoporation for 1 s, 2 s, 4 s and 8 s, cells immunoreactive for β-III tubulin constituted 76%, 75%, 85% and 78%, respectively, of GFP-transfected cells (Figure 4). These findings show that sonoporation-mediated gene transfer is effective in DRG cells.

## Discussion

Transfection of postmitotic neurons is labor-intensive, inefficient, unreliable and may have cytotoxic effects. The inability to express foreign proteins in postmitotic neurons has hampered neuroscience research [16]. Our results show that sonoporation is a feasible *in vitro *method for gene transfer into cultured DRG cells from adult rats. These results were achieved using a standard laboratory sonicator of the type used to disrupt cells and homogenize solutions. Sonoporation for 2 s at 5 W resulted in optimum transfection efficiency (31%) and a cell survival rate equivalent to 35% of that of the control.

Virus-based methods are the most successful of effecting gene transfer to neuronal cells [16,28]. The increasing use of viral vectors to transfer DNA into neurons has arisen because of their high infection efficiencies compared with nonviral methods. However, preparation of recombinant viruses is expensive and labor-intensive. Other limitations of this method are its potential toxicity for neurons, a DNA expression cassette of limited size and the production of severe immune reactions *in vivo*. These approaches also constitute a potential health hazard for laboratory personnel [29-32]. Furthermore, serious concerns about the insertional mutagenesis have arisen, especially concerning the use of viral vectors when clinical trials are involved [11,33]. By contrast, nonviral methods such as naked plasmid DNA injection, electroporation, and sonoporation should have a higher potential for clinical application, even although their efficacy for gene delivery is lower [34].

Liposome-mediated gene transfer involves the fusion of synthetic lipids into the plasmid membrane, which may affect cell membrane proteins. Therefore, sonoporation is preferable to liposome-mediated gene transfer for studying expression of transmembrane proteins. Furthermore, the efficacy of liposomal transfection is less than 1%.

Although gene transfer via electroporation *in vivo *is effective using DNA injection followed by the application of electric fields, the tissue damage caused by the electric pulse is problematic for cell survival. Ultrasound, on the other hand, makes the cell membrane porous and enhances the intracellular delivery of naked DNA *in vitro*. The membrane damage induced by ultrasound is transient and the holes (or pores) can reseal and allow survival of the cells. During sonoporation, large molecules in the medium can leak into the cells and remain trapped there after the membrane reseals [17,35]. Sonoporation has opened tremendous opportunities for targeted gene transfer. Conceptually, gene vectors mixed with ultrasound contrast agents could be injected into animal cells and targeted gene transfer could be achieved by selective application to a predefined area. Indeed, promising results have now been reported in animal models [36]. By using this approach, the risk of systemic exposure (a major drawback of current clinical gene transfer protocols) could be reduced substantially reduced.

Sonoporation of freshly dissected DRG cells was highly selective for neuronal cells. Neuronal cells constituted a much higher percentage of the total number of transfected DRG cells with the sonoporation method than with the lentivirus method. The percentage of sonoporated neuronal cells depended on energy level; under optimal sonoporation conditions, 75% of sonoporated DRG cells were neuronal cells. The mechanism underlying the preferred transfection of neuronal cells by sonoporation remains unknown. Sonoporation facilitates the entry of macromolecules into cells via microbubble-mediated cavitation and transient disruption of the plasma membrane [37,38]. As the average diameter of neuronal cells is larger than that of glial cells, it is possible that the effects of ultrasound frequency and intensity depend on cell diameter.

Sonoporation is an alternative method for transferring naked plasmid DNA into neuronal cells and may avoid side effects associated with other methods. We found that sonoporated cells maintained transgene expression for at least 2 weeks after treatment. In addition, sonoporation did not harm normal cellular functions such as the outgrowth of dendrites and axons, and all of these cell types had well-developed dendritic and axonal arbors. Neuronal cells with elaborate neurites can be transfected via sonoporation without physical disruption [39]. Thus, sonoporation does not result in genomic instability or other forms of permanent cellular damage that would limit sonoporation to short-term applications.

However, our study had several limitations. We have not evaluated a comprehensive comparison of ultrasound modalities and contrast agents. Indeed, the number of possible combinations of ultrasound field characteristics (e.g., continuous versus pulsed wave, frequency or duty cycle) and contrast microbubbles is enormous. Therefore the optimal conditions for gene transfer by sonoporation require further investigation.

## Conclusions

This study demonstrates that sonoporation enables delivery of plasmids into dorsal root ganglion cells *in vitro*. Sonoporation is a simple and economic method for studying physiological pathways in DRG cells *in vitro*. Sonoporation of dorsal root ganglion cells allows the preferential transfection of neuronal cells. Therefore, we propose that sonoporation could be applied to the intact nervous system to transfer foreign DNA for physiological research.

## Competing interests

The authors declare that they have no competing interests.

## Authors' contributions

CR carried out the all the animal studies, participated in design of the study and coordination and drafted the manuscript. KH carried out the immunohistochemistry. JT participated in participated in the design of the study and performed the statistical analysis. SH and CH carried out the cell counting. WD and YS carried out the virus preparation. All authors read and approved the final manuscript.
